# A synthetic modular approach for modeling the role of the 3D microenvironment in tumor progression

**DOI:** 10.1038/srep17814

**Published:** 2015-12-07

**Authors:** S. P. Singh, M. P. Schwartz, E. Y. Tokuda, Y. Luo, R. E. Rogers, M. Fujita, N. G. Ahn, K. S. Anseth

**Affiliations:** 1 Department of Chemical and Biological Engineering and the BioFrontiers Institute, University of Colorado at Boulder, Boulder, Colorado, United States of America; 2 Department of Biomedical Engineering, University of Wisconsin-Madison, Madison, Wisconsin, United States of America; 3 Department of Dermatology, University of Colorado School of Medicine, Aurora, Colorado, United States of America; 4 College of Medicine, Texas A&M Health Science Center, Bryan, Texas, United States of America; 5 Denver Veterans Affairs Medical Center, Denver, Colorado, United States of America; 6 Department of Chemistry and Biochemistry, University of Colorado at Boulder, Boulder, Colorado, United States of America; 7 Howard Hughes Medical Institute, University of Colorado at Boulder, Boulder, Colorado, United States of America

## Abstract

Here, we demonstrate the flexibility of peptide-functionalized poly(ethylene glycol) (PEG) hydrogels for modeling tumor progression. The PEG hydrogels were formed using thiol-ene chemistry to incorporate a matrix metalloproteinase-degradable peptide crosslinker (KKCGGPQG↓IWGQGCKK) permissive to proteolytic remodeling and the adhesive CRGDS peptide ligand. Tumor cell function was investigated by culturing WM239A melanoma cells on PEG hydrogel surfaces or encapsulating cells within the hydrogels, and either as monocultures or indirect (non-contact) cocultures with primary human dermal fibroblasts (hDFs). WM239A cluster size and proliferation rate depended on the shear elastic modulus for cells cultured on PEG hydrogels, while growth was inhibited by coculture with hDFs regardless of hydrogel stiffness. Cluster size was also suppressed by hDFs for WM239A cells encapsulated in PEG hydrogels, which is consistent with cells seeded on top of hydrogels. Notably, encapsulated WM239A clusters and single cells adopted invasive phenotypes in the hDF coculture model, which included single cell and collective migration modes that resembled invasion from human melanoma patient-derived xenograft tumors encapsulated in equivalent PEG hydrogels. Our combined results demonstrate that peptide-functionalized PEG hydrogels provide a useful platform for investigating aspects of tumor progression in 2D and 3D microenvironments, including single cell migration, cluster growth and invasion.

Tumor progression and metastasis are dependent on reciprocal changes between tumor cells and the local microenvironment, which includes genetic changes, cell-cell interactions, soluble signaling, and biochemical and biophysical extracellular matrix (ECM) properties^1^,^2^,^3^,^4^,^5^,^6^,^7^,^8^,^9^,^10^,^11^. The surrounding stroma has been implicated in regulating tumorigenesis, both in tumor suppression by healthy stroma and activation of invasion by pathogenic ECM^12^,^13^,^14^,^15^,^16^,^17^,^18^. For example, when human mammary epithelial cells were injected into cleared out mammary fat pads of mice in the presence of human fibroblastic stroma, normal breast tissue was observed^12^,^13^. Conversely, when human mammary epithelial cells were cultured without human stroma, various stages of breast cancer progression were noted, pointing to a protective effect of normal fibroblastic stroma^12^,^13^. These studies demonstrate a dual role of the stroma in tumor progression.

Two-dimensional (2D) culture surfaces, such as tissue culture polystyrene (TCP), have been instrumental for advancing our understanding of tumor biology, but planar surfaces do not recapitulate many of the complex properties of the three-dimensional (3D) *in vivo* microenvironment^2^,^3^. For example, standard TCP surfaces are undefined biochemically^19^ and have moduli several orders of magnitude higher^20^ than most tissues^21^,^22^. To address these concerns, 3D culture platforms such as collagen or Matrigel have been implemented to study tumor progression^2^,^3^. While naturally derived materials recapitulate complex features of native ECM (e.g., fibrillary structure), results are often inconsistent due to batch-to-batch variability, heterogeneity, undefined composition^23^, and gel properties that may be highly dependent on factors such as pH, temperature, or the method of extraction^24^,^25^. Further, systematic investigation of the role for specific cues on tumor progression may be confounded when using naturally derived ECM components due to endogenous bioactive amino acid sequences that are inherent to the structure of the material, as signaling moieties and matrix mechanical properties are each altered by changes in gel density. Therefore, while naturally derived materials capture important elements of the *in vivo* ECM, complementary platforms that provide greater control over materials properties would enable more systematic investigations of how the microenvironment influences tumor progression^26^,^27^.

Culture platforms based on synthetic materials may help overcome some of the limitations associated with naturally derived materials^28^,^29^,^30^,^31^, and have been applied to investigate several aspects of tumor function^26^,^32^,^33^,^34^,^35^,^36^,^37^,^38^,^39^,^40^,^41^,^42^,^43^,^44^,^45^,^46^,^47^. For example, synthetic hydrogels formed with poly(ethylene glycol) (PEG) have a high water content and are inherently bioinert, which enables researchers to introduce specific cues, such as through the incorporation of peptides that mimic full-length proteins^28^. Previous work has demonstrated that PEG hydrogels can be cross-linked with matrix metalloproteinase (MMP) sensitive peptides^48^ to enable cell induced matrix degradation and remodeling, while moieties such as the fibronectin derived adhesive peptide RGDS^49^ and others^50^ can be incorporated to promote cell-adhesion^28^,^29^,^30^,^31^, each of which play important roles during tumor progression^4^. Furthermore, the degree of cross-linking in PEG hydrogels can be tuned to achieve moduli that vary over a relatively wide range of elasticities similar to soft tissues found *in vivo*^22^,^28^,^33^,^37^. Therefore, PEG hydrogels provide an environment well suited for 3D cell culture, and one that can be tuned for desired material properties in order to imitate physiological parameters and study specific effects of the microenvironment on cell behavior^32^,^33^,^34^,^35^,^36^,^37^,^38^,^39^,^40^,^41^,^42^,^43^,^44^,^45^,^46^.

Here, we demonstrate the utility of a model synthetic platform for investigating tumor progression. Specifically, we utilize peptide functionalized PEG hydrogels^29^ formed by thiol-ene “photo-click” chemistry^51^, which has been applied to investigate a wide variety of biological questions^28^, including several aspects of tumor biology^32^,^33^,^35^,^36^,^37^,^38^,^39^,^40^,^41^,^42^. We demonstrate the versatility of thiol-ene PEG hydrogels by studying the effects of different microenvironments on migration, growth and invasiveness for the human WM239A melanoma cell line, including stromal influences on tumor function using WM239A single cells and clusters cocultured with human dermal fibroblasts (hDFs).

## Results and Discussion

**Figure 1:**
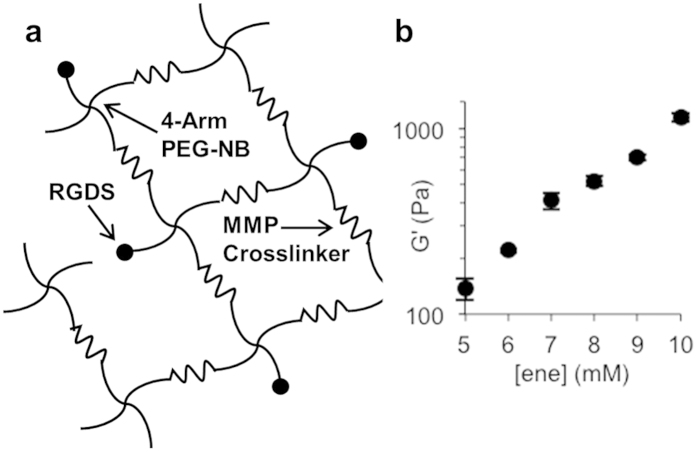
Schematic representation of poly(ethylene glycol) (PEG) hydrogels formed using “thiol-ene” chemistry. (**a**) Hydrogel networks were formed by photopolymerization to crosslink 4-arm 20 kDa PEG-norbornene (PEG-NB) molecules with an MMP sensitive peptide crosslinker (KKCGGPQG↓IWGQGCKK). An adhesive peptide moiety (CRGDS) was also added to promote cell adhesion. (**b**) Changes in shear modulus as a function of PEG-norbornene concentration (with the molar ratio of crosslinker kept constant, 1:1 thiol:ene mole ratio).

Migration, cluster growth, and invasion of a metastatic melanoma cell line (WM239A) was investigated using poly(ethylene glycol) (PEG) hydrogels to provide a “modular” platform for controlling the 3D microenvironment. Thiole-ene “photo-click” chemistry^51^ was employed to formulate PEG hydrogels (Fig. 1)^29^, in which norbornene terminated 4-arm, 20 kDa PEG was coupled with a cysteine flanked MMP sensitive peptide^48^ cross-linker as the thiol. An integrin-binding adhesion peptide (CRGDS, cysteine-arginine-glycine-aspartic acid-serine)^49^ was added to the monomer solution at 1 mM concentration for incorporation into the hydrogel network as a pendant functional group through the terminal cysteine amino acid. PEG hydrogels were formed upon exposure to 365 nm light (10 mW/cm^2^) in the presence of a photoinitiator (Fig. 1a)^52^. By varying the –ene concentration, peptide functionalized PEG hydrogels were synthesized with shear storage moduli (G’) ranging from 520–1150 Pa (Fig. 1b), which spans a range of tissues that are commonly targeted by melanoma metastases (e.g., liver, lung and brain)^21^,^22^, including lung tissue^53^, which is a metastatic site for WM239A cells^54^.

**Figure 2:**
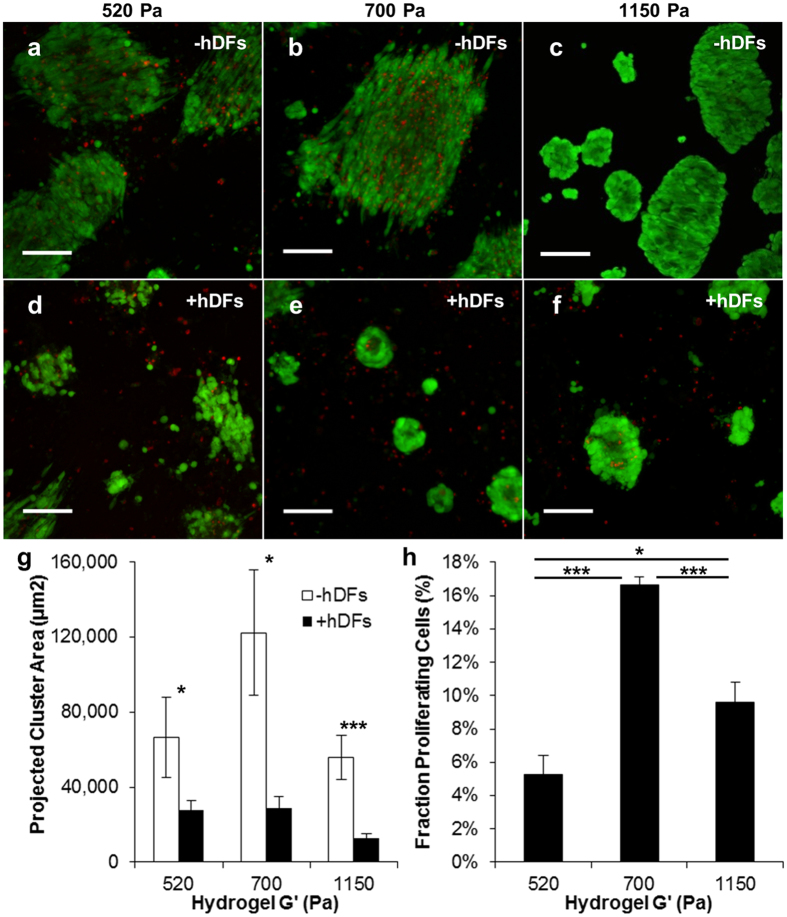
The influence of matrix modulus and stromal cells on cluster growth for WM239A melanoma cells seeded on PEG hydrogels. (**a–f**) Calcein/ethidium homodimer (Live/Dead) staining for WM239A clusters seeded on top of PEG hydrogels in (**a–c**) monoculture (−hDFs) or (**d–f**) cocultured with hDFs (+hDFs). PEG hydrogels were formed with the following shear moduli: (**a,d**) 520 Pa, (**b,e**) 700 Pa, and **(c**,**f)** 1150 Pa. Scale bars: 50 μm. (**g**) WM239A cluster size as a function of modulus after 31 days of culture on top of PEG hydrogels with (black bars) or without (white bars) hDFs (*p < 0.05; ***p < 0.001, n ≥ 20 clusters from 3 replicate hydrogels). See Supplementary Figure S1 for all statistical comparisons. (**h**) Proliferation as a function of modulus was measured by EdU assay for WM239A cells after 2 weeks of culture on PEG hydrogels (*p < 0.05; ***p < 0.001, n > 400 clusters from 3 replicate hydrogels).

It has been suggested that optimal proliferation for tumor cells correlates to sites of metastasis^55^ and tissues that support *in vivo* growth^56^. Therefore, we first investigated the growth of WM239A cells cultured on PEG hydrogel surfaces as a function of matrix modulus (520–1150 Pa; Fig. 2, Supplementary Figure S1). WM239A cells were seeded as single cells on PEG hydrogels in 24-well Transwell inserts and cultured for 31 days, during which time cells proliferated into viable cell clusters, as indicated by calcein (live)/ethidium homodimer (dead) staining (Fig. 2a–c). The relative change in cluster size was quantified by measuring projected 2D area for maximum intensity projected images from 3D z-stacks. Cluster size increased ~2-fold for WM239A cells cultured on 700 Pa hydrogels relative to softer (520 Pa) and stiffer (1150 Pa) formulations (Fig. 2g, white bars), which is in agreement with a previous study that identified a biphasic relationship between tumor cluster growth and collagen density^57^. To further examine the effect of hydrogel modulus on cluster growth, proliferation was quantified two weeks after seeding WM239A cells on top of PEG hydrogels. WM239A cells seeded on 700 Pa PEG hydrogels were characterized by rates of proliferation that were ~3-fold higher than 520 Pa hydrogels and ~1.5-fold higher than 1150 Pa hydrogels (Fig. 2h), which is consistent with results for relative cluster size (Fig. 2g).

The influence of potential stromal paracrine signaling on WM239A growth in a 2D environment was then investigated for WM239A cells cultured on PEG hydrogels and cocultured with human dermal fibroblasts (hDFs). To mimic melanoma growth before invasion into the stromal compartment^4^, single WM239A cells were first cultured for 13 days on PEG hydrogels in 24-well Transwell inserts to induce cluster formation, followed by adding hDFs encapsulated in collagen within the bottom well of the plate (see Methods for details). Collagen is the primary component of native stroma, and is widely used as a 3D scaffold for modeling hDF function^58^, and was therefore chosen as a scaffold for coculture experiments here. The extent of WM239A cluster growth was determined after 18 days of coculture with hDFs (31 days total growth). A suppression of cluster area was observed for WM239A cells cocultured with hDFs (Fig. 2d–f) relative to each monoculture condition (Fig. 2a–c), with ~3, 5, and 4-fold reductions in cluster area after 18 days of growth on 520, 700, and 1150 Pa hydrogels, respectively (Fig. 2g, black bars). Therefore, hDFs suppressed WM239A cluster growth on PEG hydrogels regardless of matrix modulus.

**Figure 3:**
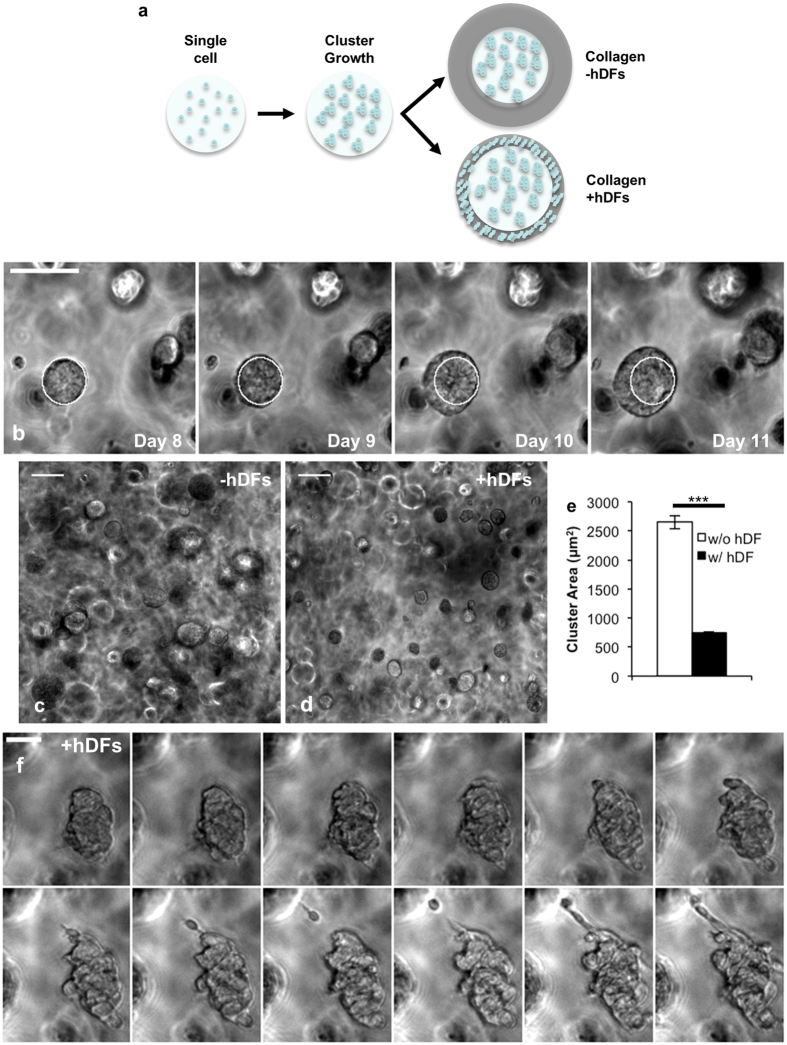
The influence of stromal cells on cluster growth and invasion for WM239A cells encapsulated in PEG hydrogels. (**a**) Schematic representation of cluster growth and coculture with hDFs. WM239A cells were encapsulated in PEG hydrogels and then surrounded by collagen with or without hDFs (+/− hDFs) on day 7. (**b**) Time-lapse images illustrating WM239A cluster growth from day 8 to day 11 (24 hrs/frame). WM239A cluster size after 11 days (**c**) as monocultures (−hDFs) or (**d**) after 7 days of monoculture followed by 4 days of coculture with hDFs (+hDFs). (**e**) Projected area for WM239A clusters cultured with or without hDFs (***p < 0.001; n > 200 clusters, ≥8 replicate hydrogels). (**f**) Time-lapse images (3 hrs/frame) beginning on day 12 for a WM239A cluster encapsulated in a PEG hydrogel and cocultured with hDFs (beginning at day 7). Scale bars: (**b–d**) 50 μm; (**f**) 25 μm.

Next, the stromal influence on WM239A cell function was investigated by encapsulating WM239A cells within PEG hydrogels. WM239A single cells were initially encapsulated in PEG hydrogels at a low density to limit cell-cell interactions, and thus the majority of clusters that formed in 3D culture represented clonal populations resulting from a single cell. WM239A cells were cultured for 7 days after encapsulation in PEG hydrogels to promote cluster growth, and then surrounded by a collagen layer that was formed either with or without hDFs (Fig. 3a). The collagen gels were formed by introducing a volume of solution that surrounded the PEG hydrogel disk, but did not cover the top surface. The PEG hydrogel was positioned in the center of the well to allow for an even collagen ring around the edges of the disk. After one day of coculture, hDFs had contracted the collagen gel to form a dense stromal region surrounding the circumference of the PEG hydrogel disk containing WM239A clusters (Fig. 3a, “Collagen + hDFs”). The collagen layer did not contract in the absence of hDFs (Fig. 3a, “Collagen -hDFs”). The projected area of WM239A clusters was ~2,600 μm^2^ after 11 days of culture in the absence of hDFs (Fig. 3b,c,e, Supplementary Movie M1). However, WM239A clusters were characterized by an ~3.7- fold lower projected area (~700 μm^2^) when co-cultured with hDFs from days 7–11 (Fig. 3d,e). Therefore, WM239A cluster growth was inhibited by coculture with primary hDFs for each of the 2D and 3D culture conditions investigated, which is consistent with a role for healthy stroma in suppressing tumor progression^12^,^13^,^14^.

Notably, many WM239A clusters transitioned to an invasive phenotype by day 12 when co-cultured with hDFs (Fig. 3f, Supplementary Movie M2), which is in agreement with previous studies demonstrating that both normal and cancer activated fibroblasts induce invasive phenotypes in tumor cells^17^,^18^,^59^,^60^. WM239A cells migrated into the surrounding regions as single cells and collectively (Fig. 3f, Supplementary Movie M2), which resembled invasion from patient-derived melanoma xenograft tumors encapsulated in equivalent PEG hydrogels (Supplementary Movie M3). WM239A single cells also became elongated and adopted motile phenotypes when cocultured with hDFs in PEG hydrogels immediately after encapsulation (Supplemental Movie M4), while monocultured melanoma cells were minimally motile and mostly migrated through a rounded mode such as previously described^41^,^42^ (Supplemental Movie M5). These combined results identify microenvironments that are useful for investigating single cell migration, cluster growth, and invasion for WM239A cells or xenograft tumors encapsulated in PEG hydrogels.

Tumor progression (tumor growth, invasion, and metastasis) depends on dynamic reciprocal interactions between cells and their surrounding 3D microenvironment^1^,^2^,^3^,^4^,^5^,^6^,^7^,^8^,^9^,^10^,^11^. Our results demonstrated that WM239A cluster size and proliferation rate were dependent on matrix stiffness for cells cultured on PEG hydrogels, and that coculture with hDFs suppressed cluster growth for each of the moduli investigated (520–1150 Pa). Cocultured hDFs also suppressed growth for WM239A clusters within PEG hydrogels, but then induced transition to an invasive phenotype characterized by both single cell and collective migration modes. WM239A cells and hDFs were not in direct contact for the models described here, which implicates soluble signaling for the suppression of cluster growth and induction of an invasive phenotype. However, we cannot rule out other factors, such as restricted mass transport or depletion of nutrients in the medium by proliferating fibroblasts. Further, the transition from growth to invasion occurred after a 4–5 day lag period when WM239A clusters were co-cultured with hDFs, which may be explained by factors other than soluble signaling, including changes in matrix properties (e.g., proteolytic degradation) or cellular phenotypes (e.g., due to genetic instability for dividing tumor cells or transition to a cancer associated fibroblastic phenotype for cocultured stromal cells). While further investigation is warranted to gain a deeper mechanistic understanding of our findings, these results demonstrate that tumor functions such as growth, migration, and invasion from both lab grown and patient-derived xenograft tumors can be mimicked using thiol-ene PEG hydrogels as the culture substrate. Our combined approach is modular in nature, allowing control over matrix properties and the timing of coculture with stromal cells, and therefore provides a versatile tool for investigating the role for several critical components of the microenvironment during tumor progression.

## Materials and Methods

### Plasmids and DNA transfection

WM239A cells were transfected with the MCAM:GFP plasmid as previously described^61^. Briefly, MCAM:GFP was made with PCR amplification of the MCAM coding region from a pSport vector (ATCC Bioproducts) containing the MCAM cDNA. The primers used were 5′-AAGCTTATGGGGCTTCCCAGGCTGGTCTCGCC and 5′- TTTGGATCCCATGCCTCAGATCGATGTATTTCTCTCC. The resulting vector was digested with BamHI and HindIII and ligated into the pEGFP-N2 vector (Clontech).

### Cell culture

WM239A melanoma cells and melanoma PDX tumors (MB947m) were cultured in RPMI 1640 (Life Technologies) supplemented with 10% Fetal Bovine Serum (Gibco) and 1% penicillin/streptomycin (Gibco). Primary neonatal human foreskin dermal fibroblasts (hDFs) were a generous gift from Prof. R. Rivkah Isseroff (University of California-Davis, Department of Dermatology)^62^. The hDFs were cultured in Dubellco’s Modified Eagle’s Medium (DMEM, Life Technologies) supplemented with 10% Fetal Bovine Serum and 1% penicillin/streptomycin.

### Hydrogel preparation and characterization

PEG norbornene (4-arm, 20 kDa)^29^, matrix metalloproteinase (MMP) sensitive cross-linking peptide (KKCGGPQG↓IWGQGCKK)^33^, cell adhesion peptide (CRGDS)^33^, and lithium phenyl-2,4,6-trimethylbenzoylphosphinate (LAP) photoinitiator^52^ were each synthesized as previously described. Stock monomer solutions were prepared in phosphate buffered saline (PBS, Gibco) and stored frozen (−80 °C). Hydrogels were polymerized using 1-[4-(2-Hydroxyethoxy)-phenyl]-2-hydroxy-2-methyl-1-propane-1-one (Irgacure 2959 or I2959) photoinitiator for cell encapsulation experiments and LAP for 2D cell seeding experiments. Stock solutions of PEG norbornene, the MMP-sensitive crosslinking peptide, CRGDS cell adhesion peptide, and LAP or I2959 photoinitiator were mixed to yield final concentrations of 1 mM CRGDS, 127 μM LAP or 2.2 mM I2959, and 6, 8, 9, or 10 mM norbornene (from 4-arm PEG-norbornene molecules) with an equivalent molar concentration of cysteines from the MMP peptide crosslinker (1:1 –ene:thiol).

### Rheometry

The monomer solution was injected into a cylindrical mold, composed of a 1 mm thick rubber gasket containing holes of 5 mm in diameter sandwiched between glass microscopy slides. This device was exposed to 365 nm light at 10 mW/cm^2^ for 90 seconds (XX-40 light source, UVP). After polymerization, hydrogels were swelled in PBS overnight to allow for equilibration. Hydrogels were placed on an ARES 4400 rheometer (TA Instruments) in a parallel plate configuration. Hydrogels were compressed 10% to minimize slippage and strain sweeps were conducted to determine the linear range of the instrument. A 20% strain was used during a frequency sweep (0.1–100 rad/s).

### Cluster growth by WM239A cells seeded on top of PEG hydrogels

A hydrogel disk was formed by pipetting 30 μL of monomer solution (8 mM/8 mM, 9 mM/9 mM, or 10 mM/10 mM -ene/thiol) into a 1 mL syringe with the tip cut off. This solution was exposed to 365 nm light (XX-40 light source, UVP) at 10 mW/cm^2^ for 90 seconds using LAP as the photoinitiator. The resulting hydrogel was placed in a 24-well plate with PBS and allowed to equilibrate for one hour. WM239A cells were trypsinized and counted with a hemocytometer. 2,000 cells were then seeded on top of each hydrogel. The cultures were maintained at 37 ^o^C at 5% CO_2_ for up to 14 days. For 2D co-culture studies, WM239A cells were allowed to attach to the hydrogel for two hours. Once attached, hydrogels were transferred to a 1 μm Transwell membrane insert (BD Falcon), inserted into a 24-well plate, and allowed to grow at 37 ^o^C at 5% CO_2_ for 13 days. On day 14, human dermal fibroblasts were trypsinized and counted. Cells were pelleted and resuspended in a 3 mg/mL PureCol® collagen (Advanced BioMatrix) solution at a cell concentration of 600,000/mL. One half mL of the resulting collagen solution was placed in the bottom of the 24-well plate containing the Transwell insert. The insert was then placed into the collagen solution and cells were again maintained at 37 ^o^C at 5% CO_2_. For control conditions, one half mL of collagen (without hDFs) was placed in the bottom of the 24-well plate. Viability and cluster size were determined by treating WM239A clusters with calcein/ethidium homodimer (Live/Dead kit, Life Technologies) using the manufacturer’s recommended protocol.

### Cell migration and cluster growth for WM239A cells cultured within PEG hydrogels

WM239A cells were trypsinized and counted with a hemocytometer. WM239A cells were pelleted and re-suspended in monomer solution at a concentration of 300,000 cells per mL for single cell migration studies and 400,000 cells per mL for cluster growth studies. A hydrogel disk loaded with WM239A cells was formed by pipetting 30 μL of 6 mM/6 mM –ene/thiol monomer solution into a 1 mL syringe with the tip cut off. The monomer solution containing single cells or the PDX tumor was exposed to 365 nm light (XX-40 light source, UVP) at 10 mW/cm^2^ for 2 minutes. Hydrogels were then transferred to a 24-well plate containing RPMI medium and maintained at 37 ^o^C at 5% CO_2_. For cluster studies, cell aggregates were grown from a single cell suspension for 7 days. After 7 days, human dermal fibroblasts were pelleted and resuspended in a 3 mg/mL PureCol® collagen (Advanced BioMatrix) solution at a cell density of 500,000 cells per mL. One half mL of the resulting collagen solution was placed in the bottom of the 24-well plate containing the hydrogel, thus surrounding the hydrogel. For control conditions, collagen without human dermal fibroblasts was pipetted into the bottom of the 24-well plate to surround the PEG hydrogel disk containing WM239A clusters. The cultures were maintained at 37 ^o^C at 5% CO_2_.

### Human melanoma patient-derived xenograft (PDX) model

The human melanoma PDX model was established as previously described^63^. Briefly, human melanoma tumors (MB947 m, lymph node metastatic tumor from superficial spreading melanoma) were obtained from a surgical specimen with written patient consent under institutional review board approved protocols at the University of Colorado Hospital, adhering to Health Insurance Portability and Accountability Act Regulations. The tumor tissue was cut into 3-mm^3^ pieces and implanted into subcutaneous pockets made by a small incision on the flank of female athymic (*nu*/*nu*) mice (NCI). Tumors were serially passaged into mice. Animal experiments were performed under the institutional guidelines for the use of laboratory animals. Human melanoma PDX tumors (MB947 m) were harvested from nude mice, diced into small pieces using a surgical blade and forceps, and encapsulated in PEG hydrogels by pipetting 30 μL of monomer solution into a 1 mL syringe with the tip cut off and manually inserting the diced tumors into the unpolymerized monomer solution, followed by polymerization as described above.

### Image analysis

#### WM239A clusters on top of PEG hydrogels

Images for calceine/ethidium homodimer labeled WM239A clusters on top of PEG hydrogels were collected on an LSM 710 confocal microscope (Zeiss). WM239A cluster size was determined from maximum intensity projection images that were converted to binary images using ImageJ^64^,^65^. The analyze particles function in ImageJ was then utilized to gather measurements of WM239A cell clusters. The relative changes in area for the tumor clusters formed on top of PEG hydrogels are an approximation of total cell number that was used to provide a simple first screen for the influences of matrix and coculture conditions on growth. Since the thickness for larger clusters increased slightly as cluster size increased, the 2D approximation represents a lower boundary for relative cell number that may slightly underrepresent the total growth rate, but does not change the qualitative conclusions drawn from the data.

#### Encapsulated WM239A clusters

Hydrogels containing encapsulated melanoma PDX or WM239A cells (or clusters) were placed in the bottom of a 24-well culture insert plate (BD Falcon) with fresh media. A Transwell insert with the membrane cut out was then used to hold the hydrogel in place. This plate was placed in an environmental control chamber (37 ^o^C at 5% CO_2_, *In Vivo* Scientific) on a Nikon TE2000-E microscope with fully automated stage position and image capture (Metamorph). Minimum intensity projections (500 μm z-stacks, 10 μm/slice) were created and converted to binary images in ImageJ^64^,^65^. The “Analyze Particles” function in ImageJ was then utilized to gather measurements of WM239A aggregates. It was assumed that the largest 2D area of the cell aggregate was representative of relative changes since there was no clear driving force in the X, Y, or Z directions. We recognize that the true growth rate may not be directly correlated to area, but the cell cluster size differences are statistically different and provide a clear measure of microenvironmental influences. Supplementary Movies and time-lapse images used for figures were drift corrected in ImageJ using the “StackReg” plugin^66^, and processed for display using the auto brightness/contrast enhancement feature.

### Proliferation Assay

WM239A cells were seeded on PEG hydrogels as described earlier. Cells were allowed to adhere to hydrogels overnight. Cells were then treated with the Click-iT EDU proliferation assay kit (Life Technologies) for 48 hours. The cells were then fixed and treated per manufacturer instructions, including DAPI staining. To image, PEG hydrogels were placed on a glass microscopy slide and a 1 mm rubber gasket, with a 5 mm hole (McMaster-Carr) was laid around the hydrogel. A glass coverslip was placed on top of the hydrogel. Immunofluorescent images of cells on PEG hydrogels were collected in a 8-bit multi track mode (wavelengths: 488 and 405) with an LSM 710 (Zeiss) confocal microscope using a 10X W Plan-Apochromat objective (1.0 DIC M27). Images were converted to a binary image and counted using the “Analyze Particles” function in ImageJ^64^,^65^.

### Statistics

Experimental averages for cell aggregate size and proliferation rate were determined as an average for all cells (at least 3 hydrogels per experimental condition). All statistical analyses were performed using a two-tailed student’s t-test. All error bars represent standard error of the mean (SEM).

## Additional Information

**How to cite this article**: Singh, S. P. *et al.* A synthetic modular approach for modeling the role of the 3D microenvironment in tumor progression. *Sci. Rep.* **5**, 17814; doi: 10.1038/srep17814 (2015).

## Supplementary Material

Supplementary Information

Supplemental Movie M1

Supplemental Movie M2

Supplemental Movie M3

Supplemental Movie M4

Supplemental Movie M5
