# Supplementary material for: A synthetic modular approach for modeling the role of the 3D microenvironment in tumor progression
Source: Sci Rep. 2015 Dec 7;5:17814. doi: 10.1038/srep17814 (PMC4671067; doi:10.1038/srep17814)
Supplement: Supplementary Information [file srep17814-s1.pdf]

A synthetic modular approach for modeling the role of the 3D microenvironment in tumor progression

S.P. Singh[1], M.P. Schwartz[2], E.Y. Tokuda[1], Y. Luo[3], R.E. Rogers[4], M. Fujita[3,5], N.G. Ahn[6], and K.S. Anseth[1,7,*]

[1]Department of Chemical and Biological Engineering and the BioFrontiers Institute, University of Colorado at Boulder, Boulder, Colorado, United States of America

[2]Department of Biomedical Engineering, University of Wisconsin-Madison, Madison, Wisconsin, United States of America

[3]Department of Dermatology, University of Colorado School of Medicine, Aurora, Colorado, United States of America

[4]College of Medicine, Texas A&M Health Science Center, Bryan, Texas, United States of America

[5]Denver Veterans Affairs Medical Center, Denver, Colorado, United States of America

[6]Department of Chemistry and Biochemistry, University of Colorado at Boulder, Boulder, Colorado, United States of America

[7]Howard Hughes Medical Institute, University of Colorado at Boulder, Boulder, Colorado, United States of America

* kristi.anseth@colorado.edu

Keywords. tumor modeling, melanoma, proliferation, paracrine signaling, cell-cell signaling, microenvironment

		-hDFs			+hDFs	
		520 Pa	700 Pa	1150 Pa	520 Pa	700 Pa
**-hDFs**	700 Pa	0.16413				
	1150 Pa	0.70508	0.10634			
**+hDFs**	520 Pa	0.03713	0.00081	0.01428		
	700 Pa	0.05011	0.00110	0.03452	0.88769	
	1150 Pa	0.00559	0.00029	0.00003	0.02035	0.03488

Supplementary Figure S1. Statistical comparisons for WM239a cluster growth on PEG hydrogels. A student's *t*-test was used to determine statistical significance for comparisons between conditions (graphs shown in Figure 2).

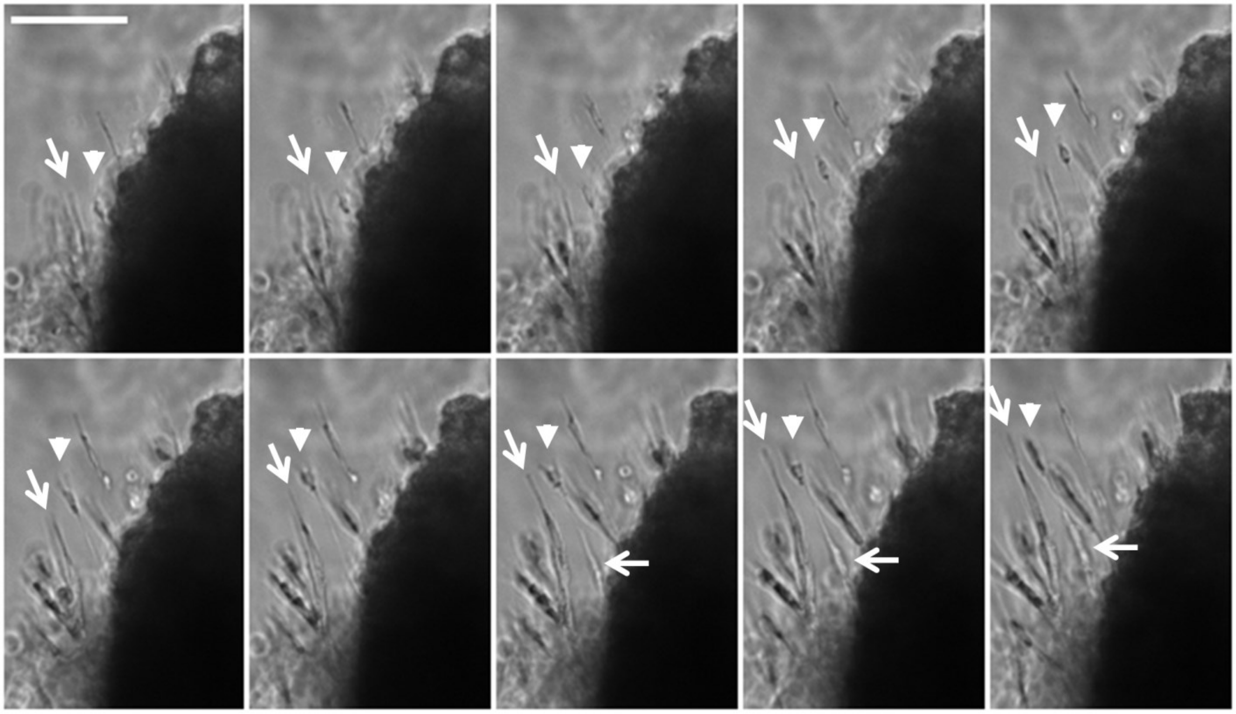

Supplementary Figure S2. Invasion from a human melanoma patient-derived xenograft (PDX) tumor encapsulated in a PEG hydrogel. Time-lapse microscopy (1 hr / frame) illustrating invasion from a human melanoma PDX tumor (MB947m) encapsulated in a PEG hydrogel. Both single cell (arrowheads) and collective (arrow) migration modes were observed. **Scale bar** = 50 μm.

Supplementary Movie Captions

Supplementary Movie M1. WM239A cluster growth in a PEG hydrogel. WM239a single cells were encapsulated in a PEG hydrogel and cultured for 7 days to initiate cluster formation. The PEG hydrogel was then surrounded by collagen (without hDFs) and cultured overnight before imaging (3 hrs / frame). Scale bar = 50 μm.

Supplementary Movie M2. Single cell and collective migration from WM239a clusters cultured in a PEG hydrogel and surrounded by hDFs in collagen. WM239a single cells were encapsulated in a PEG hydrogel and cultured for 7 days to initiate cluster formation. The PEG hydrogel was then surrounded with hDFs encapsulated in collagen and cultured for an additional 4 days before imaging (3 hrs / frame). Scale bar = 25 μm.

Supplementary Movie M3. Single cell and collective migration from a human melanoma patient-derived xenograft (PDX) tumor encapsulated in a PEG hydrogel. A human melanoma PDX tumor (MB947m) was encapsulated in a PEG hydrogel and cultured for 24 hours before imaging (30 min / frame). Scale bar = 50 μm.

Supplementary Movie M4. Migration for WM239A single cells encapsulated in a PEG hydrogel and surrounded by hDFs in collagen. WM239A cells were encapsulated in a PEG hydrogel and immediately surrounded with hDFs encapsulated in collagen. Time-lapse imaging began 24 hours after encapsulation (1 hr / frame). The white line indicates the boundary between the PEG hydrogel and collagen. Scale bar = 50 μm.

Supplementary Movie M5. Migration for WM239A single cells encapsulated in a PEG hydrogel. WM239A cells were encapsulated in a PEG hydrogel (without hDFs). Time-lapse microscopy began 24 hours after encapsulation (30 min / frame). Scale bar = 50 μm.
